# Directed altruistic living donation: what is wrong with the beauty contest?

**DOI:** 10.1136/medethics-2014-102230

**Published:** 2015-06-30

**Authors:** Greg Moorlock

**Keywords:** Transplantation, Allocation of Organs/Tissues, Donation/Procurement of Organs/Tissues

## Abstract

This paper explores the specific criticism of directed altruistic living organ donation that it creates a ‘beauty contest’ between potential recipients of organs. The notion of the beauty contest in transplantation was recently used by Neidich *et al* who stated that ‘[a]ltruism should be the guiding motivation for all donations, and when it [is], there is no place for a beauty contest’. I examine this beauty contest objection from two perspectives. First, I argue that, when considered against the behaviour of donors, this objection cannot be consistently raised without also objecting to other common aspects of organ donation. I then explore the beauty contest objection from the perspective of recipients, and argue that if the beauty contest is objectionable, it is because of a tension between recipient behaviour and the altruism that supposedly underpins the donation system. I conclude by briefly questioning the importance of this tension in light of the organ shortage.

## Introduction

This paper will explore the specific criticism of directed altruistic living organ donation (DALD) that it creates a ‘beauty contest’ between potential recipients of organs. The notion of the beauty contest in transplantation was recently used by Neidich *et al* who stated that ‘[a]ltruism should be the guiding motivation for all donations, and when it [is], there is no place for a beauty contest’ (p.43).^1^ I will examine this beauty contest objection from the perspective of donors and recipients, and will argue that the potential conflict with altruism does not, as may often be thought, arise from the actions of the donors, but rather from the behaviour of the potential recipients. I will argue that whether this poses a problem for DALD rests on the disputed role of altruism in organ donation.

## Background

There is a shortage of organs available for transplantation in the UK, so patients often have to endure long periods of illness before they eventually receive a transplant, or sometimes they die before being offered a transplant. There are two overarching approaches to increasing the number of donated organs: increasing deceased donations or increasing living donations. Increasing the number of deceased donations can be achieved by improving consent rates and effectively using poorer-quality organs. Increasing the number of living donors is more difficult because it relies on healthy individuals putting themselves forward for major surgery to benefit others.

Historically, living donation was only permitted when the donor and recipient were either genetically related or in an appropriate qualifying relationship.^2^ This changed in 2007 when the first non-directed altruistic living donation (N-DALD) was permitted, and a healthy donor gave a kidney to a stranger on the waiting list. N-DALD organs are allocated according to the same impartial criteria used for deceased donation, or used to create paired/pooled transplant chains.^3^ Although there was some initial resistance to the notion of N-DALD,^4^ it is now an accepted source of good-quality, if few in number, donations, which have saved additional lives.^5^

More recently, DALD has been cautiously accepted in the UK. This allows donors to direct their altruistic donations to specific recipients, even though there may be no pre-existing relationship between them. This has occurred in the USA since 2004 via dedicated websites such as matchingdonors.com, but has only been permitted in the UK since 2013. In the USA, matchingdonors.com charges potential recipients a registration fee, but any kind of commercial transaction, including registration fees, renders DALD ineligible in the UK. Potential donors join the website (free of charge), look through potential recipients and choose a potential candidate for their organ. As well as registering on dedicated websites, patients can advertise for potential donors via social media such as Facebook, Twitter or Youtube. Although there is flexibility in the finer details of how DALD might operate, this paper will concentrate primarily on the use of donor/recipient-matching websites such as matchingdonors.com.

### The adverts

Many patients’ adverts for donors feature videos, photos and text outlining their medical condition and their (frequently bleak) prognosis without a transplant. This content seems unobjectionable, but many adverts go far beyond this. Some videos/photos include not just the patient, but also their friends and family. These people will provide testimonials explaining why a donor should choose that specific person to donate an organ to. Patients are frequently shown surrounded by their young families, with their children explaining how desperately they need their parent to receive a transplant. While this pointedly emphasises the importance of donating organs, it also has a potentially less desirable effect: organ donation becomes a form of beauty contest where patients with the most heart-rending story are most likely to receive transplants. Potential recipients are also keen to demonstrate moral worthiness: many list the charitable activities that they support, emphasise how active they are within their community or local church, and highlight that their illness is not their fault. Trying to influence people's donation decisions with information like this represents a significant departure from the usual impartial allocation procedure for altruistically donated organs.

### Policy position

DALD facilitated by websites has met with resistance in the USA^6^ ^7^ and the UK.^8^ The Human Tissue Authority regards DALD as legal provided there is no evidence of reward or coercion.^9^ There is, apparently assumed to be, an increased likelihood of coercion or reward with DALD in comparison to living-related donations, so the assessment process for potential donors is particularly rigorous. The British Transplant Society suggests that non-directed altruistic donation is preferable to DALD, as it preserves equity of access.^10^ They, therefore, suggest that the possibility of non-directed donation should be discussed with all potential DALD donors. In short, DALD appears to be tolerated rather than promoted in the UK, unlike N-DALD, which is celebrated.^11^ The dire consequences of the organ shortage mean that one should have good reasons for failing to take full advantage of a particular source of donations. There may be some general concerns over promoting living donation when there are still potential gains to be made in deceased donation rates (given that living donation involves subjecting a healthy person to surgery and deceased donation does not), but the current reluctance to embrace and promote DALD to lessen the organ shortage appears to be grounded in more specific concerns.

### The beauty contest criticism

The existing literature discussing donor/recipient-matching websites suggests that the primary objections are increased risk of reward or coercion,^6^ ^7^ and the beauty contest dynamic, which results in allocation contrary to medical criteria.^1^ ^7^ These objections seem reasonable, but concerns about reward and coercion can be somewhat allayed by the introduction of appropriately rigorous safeguarding and screening.

A further ethical criticism is that of the beauty contest. By this, one need not mean that literally the most beautiful candidates are selected, but rather that recipients are selected from a list according to criteria that make them an attractive choice for donors. Many of these criteria are not obviously ‘medical’. For instance, choosing a particular recipient because they created a funny video, or because they have young children who desperately need a parent would clearly deviate from the usual ideas of urgency and medical need. The idea of a beauty contest to determine who lives and who dies certainly seems distasteful,^i^ but distasteful is not always unethical. Neidich *et al* have suggested that the beauty contest dynamic is unacceptable because it is not compatible with altruism. I will now explore this claim from the perspective of donors and recipients, and will argue that if beauty contests in organ donation represent a failure of altruism on the part of donors then this is not unique to DALD. I will then explore the issue from the perspective of recipients, and will argue that it is their actions that are at odds with an altruistic donation system.

## Beauty contest and the donors

Neidich *et al* suggest that sites such as matchingdonors.com ‘move beyond conventional directed donation [to friends or family members] because there is more than one identified patient in need’ (p.44).^1^ This creates the beauty contest dynamic, which allows donors to shop for a winner. If browsing for a winner is objectionable, it may be either that choosing between recipients is itself objectionable, or that the criteria open to use are potentially objectionable. Choosing between recipients per se is plainly not objectionable; members of transplant staff do this all the time, and it is necessary in any transplant system operating in a situation of shortage. One might instead suggest that it is wrong for *donors* to choose, but this already occurs in the directed donations to friends or family members that are considered uncontroversial. Most people who know someone needing a kidney transplant will also be aware that there are lots of other, although unidentified, people who similarly require a transplant. A potential donor may choose to donate a kidney to his/her friend/relative over the unidentified strangers. Living donors in the UK are asked when they donate whether, if it transpires that their donated organ is not suitable for the intended recipient, they are happy for it to be allocated to the general pool. Many will presumably say yes, but in doing so they clearly state their preference that the organ is allocated to their chosen recipient in the first instance. This is a case of choosing an identified person over an unidentified person on non-medical grounds. The situation does not necessarily seem less acceptable, however, if a person chooses between identified people. For instance if a person knows three people who need transplants—a best friend, a work colleague and someone on a donor-matching website—it is not obviously unacceptable that he/she could choose one over the others, even if this is on the basis of non-medical criteria such as being good friends with the needy patient.

Neidich *et al* appear to be concerned about organs being allocated according to what they term ‘normative classifications’, including clinical diagnosis, race or religion (p.44).^1^ Unfortunately, they do not explain what they mean by normative classification, but one presumes that they mean something other than just organs being allocated according to non-medical criteria: I could quite acceptably donate a kidney to a close friend, even if there were more urgent cases or better matches on the waiting list. Classifying people into friends or non-friends is a normative classification, although one that is generally considered acceptable. Through a website, however, donors could be choosing specific recipients because they are of a certain ethnic origin, for example. Maybe, then, some normative classifications reflect unacceptable prejudice. These objectionable normative classifications can appear elsewhere within organ donation, however; so, it would be inconsistent to single out DALD as unacceptable. Just as someone might choose a recipient because of certain normative classifications, people are free to choose their friends or spouses on the same grounds—for instance some people will only marry members of the same religion. If normative classifications shape who one is friends with then they also play an influential role in many cases where one chooses to donate an organ to a friend, and there is no guarantee that these will be ‘acceptable’ normative classifications.

Neidich *et al*'s more general concern appears to hinge on the suggestion that the beauty contest dynamic is not compatible with the altruism required from donors. They suggest that altruism, which is supposed to motivate organ donation, in its purest form dictates that ‘there is no shopping among patients; rather there is a donation to any patient with the capacity to benefit’ (p.45).^1^ Theoretical doubts about the operation of altruism within organ donation have already been raised,^13^ ^14^ but altruism still seems to be regarded by some as a necessary requirement of ethical donation.^1^ Neidich *et al*'s suggestion that the purest form of altruism (which presumably requires a lack of self-interest, so that the act is disinterestedly other-regarding) prohibits shopping among patients seems rather dubious, however. Insistence on a pure account of altruism might still allow, or even *require,* a donor to shop between potential recipients to ensure that his/her altruism is directed towards the right person (where this is defined in agent-neutral terms, and may not correspond with the ‘right’ person as judged by medical criteria). For instance, someone truly concerned about altruism and helping other people might want to ensure that his/her donated organ is given not just to anyone, but to someone who will also help other people: this seems *more* altruistic, rather than less. The Nuffield Council on Bioethics considered altruism in some detail in a report on donation of bodily materials in the UK (including organs), and their definition of altruism (p.139)^15^ focuses on selflessness and lack of anticipated reward, and so says nothing that obviously prohibits choosing recipients. Moreover, insisting that organ donation is only ethical if it is motivated by pure altruism will pose problems for directed donation to family members, which is difficult to conceive of as *purely* altruistic. In other contexts, motivation other than purest altruism is considered an acceptable reason to donate to a cause, so to claim that organ donation has some privileged requirement of purity would require further justification. If I choose to donate £10 000 000 to a testicular cancer charity rather than a breast cancer charity solely because I am a male and men's health is of more direct concern to me, it would seem very demanding to suggest that my potentially life-saving donation was unethical and should not be permitted.

It seems that it is challenging to robustly criticise DALD and beauty contests from the perspective of donors without introducing unjustifiable inconsistency. Pure altruism is not required in other aspects of organ donation, so, it would be inconsistent to require it within DALD. We routinely let donors express preference based on the normative classification that ‘someone is my friend/relative’, so, an outright ban on allocation according to normative classifications would be difficult. If the beauty contest dynamic is objectionable, it must be for other reasons.

## Beauty contest and the recipients

I have argued thus far that the beauty contest criticism is not wholly convincing when considered from the perspective of donors. I will now explore the beauty contest dynamic from the perspective of recipients.

### Receiving organs as a competition

Waiting lists are the means used in the UK to manage the shortage of donated organs. Factors considered when making allocation decisions vary depending on the type of organ, but it is not a simple case of first-come first-served.^16^ The Oxford English Dictionary defines ‘competition’ as ‘the striving of two or more for the same object’,^17^ so, in a simple sense, those who are waiting for an organ transplant are competing against each other. Each potential recipient on the waiting list is considered against the relevant allocation criteria, and when an organ becomes available, the patient at that time with the highest ‘score’ relative to that organ will be offered it.

DALD may create a beauty contest dynamic, which is plainly competitive, but if, as suggested here, competition in a simplistic sense exists in transplantation outside of DALD, one must suggest why some forms of competition are considered acceptable and others are not.

### Contrasting types of competition

When organs are donated via N-DALD, or the majority of deceased donation, the rules of the competition are straightforward. Organs are allocated according to criteria that aim to balance waiting-list mortality and transplant outcomes.^18^ Although these criteria may sometimes work to the detriment of specific individuals, they balance the competing concerns of the overall system that operates in the context of a welfare state. Patients who wait to receive organs via this approach to allocation may accept that although they may have to wait, those with the most urgent need that can be effectively met will be given priority, and that should their need become urgent, they will be afforded this same priority. Although this may still be competition by definition, this approach can also be reasonably characterised as being cooperative. They are all relying on the same system for rescue from their plight, and patients who rely on these systems to obtain an organ are not actively competing by trying to gain priority for themselves, but are instead waiting their turn on the basis of the rules of allocation.

In contrast to the orderly cooperation of impartial allocation, the competition for organs via DALD is significantly more active: the behaviour of recipients can directly influence the likelihood of them receiving a transplant. Instead of patiently waiting to move to the top of a waiting list due to being the best match for an organ, patients can increase their chances of being offered a transplant by creating the most compelling back-story, creating the best viral video or by directly contacting the greatest number of potential donors. By competing for organs actively and trying to get to the top of each prospective donor's ‘list’, patients vying for organs via DALD may give little concern to the plight of others who also need transplants. By taking the attitude of ‘please give an organ to me rather than anybody else’, potential recipients place their own interests ahead of others with potentially much more urgent need. The consequences of this will vary, but in some cases, it may result in people with urgent need dying before a transplant becomes available or having to endure extended periods of increasingly greater suffering.

This may not always seem wrong—if one is in desperately urgent need of a life-saving resource, and is offered such a resource, it would be very demanding to insist that one is morally bound to pass the resource onto someone who has marginally more urgent need for that resource, particularly if it is unlikely that any more of that resource will become available. In many cases, however, the disparity in urgency of need will be much greater. Someone who could wait 2 years for a transplant trying to place themselves ahead of someone who can only wait 2 weeks (where they are both competing for the same donor via a website) is akin to a strong swimmer trying to push a very weak swimmer away from a lifeboat even though another lifeboat will probably eventually return to pick up the strong swimmer anyway.

The competition introduced by DALD is potentially complex. While some people who may have, otherwise, donated via N-DALD may now choose to donate to a specific recipient, there may be some donors who would only be motivated to donate by the situation of a single recipient, and would not have donated via N-DALD. In these cases, those recipients should not be considered to be competing with anybody—in fact, their actions here may help others, insofar as they attract a donor who would not be willing to donate to others and remove a patient from the waiting list^ii^ (much like living-related donation). Despite this, it does seem plausible that DALD increases the likelihood of active competition at two levels. First, if people who would have previously donated via N-DALD now consider donating via DALD then DALD recipients are actively competing with N-DALD recipients. Second, if potential donors visit donor-matching websites with a relatively open mind about to whom to donate, DALD recipients are actively competing with one another.

It is here that there is scope for objection to the organ donation beauty contest on altruistic grounds. I do not wish to claim that recipients are duty-bound to give consideration to other people on the waiting list in all situations, as this may be supererogatory in many instances. But if potential recipients are asking others to be moved to altruism by their need for a transplant and the person that they are, it is jarringly self-regarding for recipients to then fail to consider the situations and qualities of similar others. These recipients are asking others to do for them what they themselves are not willing to do for others. It would be difficult to defend the claim that a recipient ought to act altruistically, but this is not necessary. Instead, I wish to make the more modest claim that if it is important for a donation system to be driven by altruism, the behaviour of recipients should at least not counteract the altruistic underpinnings of such a system. The precise role of altruism in organ donation is subject to debate, but the Nuffield Council on Bioethics has stated that altruism ‘helps underpin a communal, and collective, approach to the provision of bodily material for others’ needs, where generosity and compassion are valued’ (p.132),^15^ and similar claims have been made by others (p.113).^14^ The behaviour of recipients in impartial allocation systems who cooperatively wait their turn is aligned with the altruistic basis of the system because the waiting list is ordered to provide the best overall balance for everyone waiting for organs. By accepting that organs are allocated according to the waiting list order, patients allow for their needs and interests to be considered against the needs and interests of other patients to ensure that overall need is effectively met. The behaviour of many recipients in DALD, however, can work against the role of altruism because those taking an ‘every person for him/herself’ approach would likely be much more self-serving. Rather than embracing a communal and collective approach to meeting the needs of those who require transplants, DALD allows recipients to actively further their own interests by leveraging their personal appeal to place their needs above those of others, potentially at a significant cost to those with more urgent need.

## The empirical questions and the (un)importance of altruism

I have examined a specific criticism of beauty contests dynamics within organ donation, and have not given much consideration to the potential justice issues of such a dynamic. This is partly because many arguments have been explored already in the context of directed deceased donation,^19^ and also because the consequences are so unpredictable. The matchingdonors.com website claims to have matched over 300 recipients since its creation,^20^ so has provided a tiny proportion of organ donations in the USA. It is not obvious what its impact would be in the UK, but one might expect similarly low numbers unless it was extensively pushed as a means of promoting donation. What is especially unknown is its impact on N-DALD: for example, would people, who would otherwise donate impartially, now choose to donate via DALD? For reasons of efficiency, impartial organ donation may be preferable to partial donation, as it allows for organs to be given to the most medically appropriate patients. If donor/recipient-matching websites cause people who would, otherwise, choose to donate non-directedly to donate directedly then this is an undesirable consequence of these sites.

Neidich *et al*^1^ and others^6^ ^7^ are wise to raise concerns about the potential for injustice to occur as a result of DALD, but the scale and impact of this is unknown. One possible outcome is that DALD causes people to donate who, otherwise, would not do, and provides additional benefit to transplant patients. Even if this benefit is distributed unevenly on unjustifiable grounds, assuming these donations would not have happened without DALD then accepting them makes nobody worse off.^21^ It is with this consideration in mind that decisions should be made about DALD and the future of organ donation more generally. Neidich *et al* are correct to assert that aspects of DALD are not compatible with altruism, although I have argued that this failing lies more with recipients than donors. DALD may, therefore, conflict with organ donation's pure (and often incorrect) self-image of altruistic gifting. If altruism is *the* ethical guideline of acceptable organ donation then DALD via websites seems at odds with this principle, and should not be permitted.

But if, as has been suggested elsewhere,^13^ ^22^ altruism may not be a necessary condition of acceptable organ donation then preventing extra donations because they do not comply with the altruistic self-image of organ donation could be looking a gift horse in the mouth. A donation and transplantation system underpinned by altruism, cooperation and selflessness may seem like the ideal, but clinging onto this ideal seems questionable if it actually results in less good being done via transplantation than the alternatives.

## Footnotes

^i^One may recall a hoax Dutch television programme that caused controversy when it pretended that viewers could vote on which patient should be given a transplant.^12^

^ii^This point was made by an anonymous reviewer, to whom I am grateful.

## References

[1] Neidich EM, Neidich AB, Cooper JT, et al The ethical complexities of online organ solicitation via donor–patient websites: avoiding the “beauty contest”. Am J Transplant 2012;12 (1):43–7. 10.1111/j.1600-6143.2011.03765.x 21951635

[2] Human Tissue Authority. Code of practice 2: living organ donation. http://www.hta.gov.uk/legislationpoliciesandcodesofpractice/codesofpractice/code2donationoforgans.cfm?FaArea1=customwidgets.content_view_1&cit_id=673&cit_parent_cit_id=669 (accessed 3 Mar 2015).

[3] NHS Blood and Transplant. Non-directed altruistic donation. http://www.organdonation.nhs.uk/how_to_become_a_donor/living_donation/national_living_donor_kidney_sharing_scheme/non-directed_altruitic_donation/ (accessed 3 Mar 2015).

[4] Kaplan BS, Polise K In defense of altruistic kidney donation by strangers. Pediatr Nephrol 2000;14 (6):518–22. 10.1007/s004670050806 10872197

[5] NHS Blood and Transplant. Kidney activity. https://nhsbtmediaservices.blob.core.windows.net/organ-donation-assets/pdfs/kidney_activity.pdf (accessed 3 Mar 2015).

[6] Truog RD The ethics of organ donation by living donors. N Engl J Med 2005;353 (5):444–6. 10.1056/NEJMp058155 16079366

[7] Wright L, Campbell M Ethical issues in dialysis: soliciting kidneys on web sites: is it fair? Semin Dial 2006;19 (1):5–7. 10.1111/j.1525-139X.2006.00110.x 16423173

[8] British kidney donors select their own recipient through controversial website. *ITV News*, 29 Aug 2012. http://www.itv.com/news/2012-08-29/uk-donors-select-a-patient-after-emotional-online-appeal/ (accessed 3 Mar 2015).

[9] Human Tissue Authority. *Guidance to Transplant Teams and Independent Assessors* 2012 https://www.hta.gov.uk/sites/default/files/Guidance_to_Transplant_Teams_and_Independent_Assessors.pdf (accessed 3 Mar 2015).

[10] British Transplantation Society. *Guidelines for Directed Altruistic donation* 2013 https://www.bts.org.uk/Documents/Guidelines%20for%20Directed%20Altruistic%20Donation-%20Final%20Version%2004-2013.pdf (accessed 3 Mar 2015).

[11] Human Tissue Authority. *Huge Rise in Altruistic Donor Numbers* 2013 http://www.hta.gov.uk/newsandevents/htanews.cfm/1143-Huge-rise-in-altruistic-donor-numbers--says-HTA.html (accessed 3 Mar 2015).

[12] NHS Blood and Transplant. Dutch TV Show. http://www.organdonation.nhs.uk/newsroom/statements_and_stances/statements/dutch_tv_show.asp (accessed 3 Mar 2015).

[13] Moorlock G, Ives J, Draper H Altruism in organ donation: an unnecessary requirement? J Med Ethics 2014;40 (2):134–8. 10.1136/medethics-2012-100528 23538329 PMC3913211

[14] Hinkley C Moral Conflicts of Organ Retrieval: A Case for Constructive Pluralism. Amsterdam: Radopi, 2005.

[15] Nuffield Council on Bioethics. Human bodies: donation for medicine and research. London: Nuffield Council on Bioethics, 2011.

[16] NHSBT. Organ allocation. http://www.organdonation.nhs.uk/about_transplants/organ_allocation/index.asp (accessed 3 Mar 2015).

[17] Oxford English Dictionary, Entry for ‘Competition’, http://www.oed.com/view/Entry/37578?rskey=don97n&result=1&isAdvanced=false#eid (accessed 3 Mar 2015).

[18] Neuberger J, Gimson A, Davies M, et al Selection of patients for liver transplantation and allocation of donated livers in the UK. Gut 2008;57 (2):252–7. 10.1136/gut.2007.131730 17895356

[19] Wilkinson TM Ethics and the acquisition of organs. Oxford: Oxford University Press, 2011.

[20] MatchingDonors.com Newsletter. *Matching Donors*, 8–12 Jun 2015.. http://www.matchingdonors.com/newsletter.cfm (accessed 6 May 2014).

[21] Wilkinson TM Racist organ donors and saving lives. Bioethics 2007;21 (2):63–74. 10.1111/j.1467-8519.2007.00526.x 17845490

[22] Saunders B Altruism or solidarity? The motives for organ donation and two proposals. Bioethics 2012;26 (7):376–81. 10.1111/j.1467-8519.2012.01989.x 22827319

